# Impact of subgingival periodontal treatment on systemic markers of inflammation in patients with metabolic syndrome: a systematic review of randomized clinical trials

**DOI:** 10.3389/froh.2024.1465820

**Published:** 2025-01-20

**Authors:** Marie Chavez, Asshly Ramirez, Akram Hernández-Vásquez, Daniel Comandé, Diego Azañedo

**Affiliations:** ^1^ Universidad Científica del Sur, Lima, Peru; ^2^ Centro de Excelencia en Investigaciones Económicas y Sociales en Salud, Vicerrectorado de Investigación, Universidad San Ignacio de Loyola, Lima, Peru; ^3^ Instituto de Efectividad Clínica y Sanitaria (IECS), Buenos Aires, Argentina

**Keywords:** subgingival periodontal treatment, antibiotic therapy, systemic markers of inflammation, metabolic syndrome, randomized clinical trials, systematic review

## Abstract

**Introduction:**

This study synthesizes evidence on the impact of subgingival periodontal treatment combined with antibiotics on reducing systemic inflammation markers—C-reactive protein (CRP), interleukins, and tumor necrosis factor-alpha (TNF-α)—in patients with metabolic syndrome (MS) and periodontal disease (PD), compared to supragingival periodontal treatment with placebo.

**Methods:**

Randomized clinical trials (RCTs) published in English, Spanish, or Portuguese that addressed the research question were included. A search was conducted in eight databases (PubMed, EMBASE, CINAHL, LILACS, Scopus, WoS Core Collection, Dentistry & Oral Science Source, and Cochrane Central) on June 20, 2023. Risk of bias was assessed using the Cochrane RoB 2 tool, and evidence certainty was evaluated following GRADE guidelines. A qualitative synthesis of the evidence was performed.

**Results:**

Two RCTs with 228 participants (ages 35–65) were included. Montero et al. reported significant reductions in CRP levels favoring the treatment group at 3 months (2.7 mg/L ± SE: 0.4 vs. 3.9 mg/L ± SE: 0.6; *p* = 0.001) and 6 months (2.9 mg/L ± SE: 0.4 vs. 4.0 mg/L ± SE: 0.8; *p* = 0.004). Lopez et al., however, found no significant differences throughout follow-up. Only Montero et al. reported on interleukin 1β and TNF-α, observing significant reductions at 3 months for interleukin 1β (0.9 pg/dl ± SE: 0.1 vs. 2.3 pg/dl ± SE: 0.5; *p* = 0.046) and TNF-α (6.4 pg/dl ± SE: 0.8 vs. 10.0 pg/dl ± SE: 2.3; *p* = 0.037).

**Discussion:**

The evidence is limited by the small number of comparative RCTs. One RCT with low risk of bias demonstrated significant reductions in CRP, interleukins, and TNF-α levels at 3 months and CRP at 6 months. The other, with unclear risk of bias, showed no differences in CRP up to 12 months. Findings suggest that subgingival periodontal treatment with antibiotics reduces systemic inflammation for up to 6 months in patients with MS and PD. However, larger RCTs with standardized methods and longer follow-up are needed to confirm these results.

**Systematic Review Registration:**

https://www.crd.york.ac.uk/prospero/display_record.php?ID=CRD42022366056, PROSPERO (CRD42022366056).

## 1 Introduction

Periodontal disease is a common condition in patients with metabolic syndrome (1). Metabolic syndrome is a set of metabolic alterations, which include the presence of central obesity, decreased high-density lipoprotein (HDL) cholesterol, altered triglyceride (TG) concentrations, hypertension and hyperglycemia (2). In periodontal disease, the epithelium of the subgingival pocket is inflamed and ulcerated, facilitating the entry of various pathogenic microorganisms from dental plaque, leading to bacteremia (3). This condition triggers the production of inflammatory markers, such as interleukin-1-beta (IL-1β), tumor necrosis factor-alpha (TNF-α), interleukin-6 (IL-6) and prostaglandin E2 (PGE2). These markers can spread to the bloodstream and trigger a systemic inflammatory response (3). Periodontal disease may also increase the risk of a cardiovascular event as recurrent bacteremia with periodontal pathogens can damage vascular endothelium and smooth muscle cells leading to atherosclerosis (4). Therefore, chronic systemic inflammation generated as a consequence of metabolic syndrome and periodontal disease may be a common factor between these two diseases, leading to an increased risk of developing atherosclerosis (5).

Evidence suggests that subgingival periodontal treatment, together with systemic antibiotics, in patients with metabolic syndrome and periodontal disease, could improve the systemic inflammation present in these patients, which would be evidenced by a reduction in markers such as C-reactive protein (CRP), interleukins, and TNF-α (5, 6). Thus, for example, one of the randomized clinical trials (RCTs) published on the subject reported that receiving intensive periodontal treatment (IPT) (scaling and root planing plus azithromycin 500 mg every day for three days) in patients with metabolic syndrome and severe periodontitis was associated with significant reductions in CRP, IL-1β, and TNF-α levels for up to 6 months of follow-up (6). However, to date there is no systematization of the evidence regarding the efficacy of these therapies in these patients.

The aim of this study was to determine the impact of subgingival treatment plus antibiotic therapy on the reduction of systemic markers of inflammation in patients with metabolic syndrome and periodontal disease by means of a systematic review of RCTs. The evidence generated could be useful for clinical decision making in the management of patients with metabolic syndrome and periodontal disease, and could also serve to identify gaps in knowledge for future research on the subject.

## 2 Methods

The present study is a systematic review of RCTs. The search protocol was registered in PROSPERO (CRD42022366056), and the present manuscript follows the guidelines of the PRISMA (*Preferred Reporting Items for Systematic Reviews and Meta-Analyses*) statement for reporting (7).

### 2.1 Eligibility: inclusion and exclusion criteria

We included original articles from RCTs, that addressed the components of the research question as follows:

P: Patients with metabolic syndrome and periodontal disease.
I: Subgingival periodontal treatment (scaling and root planing) + antibiotics.
C: Supragingival scaling treatment + placebo.
O: Systemic markers of inflammation [CRP [mg/L], interleukins [pg/dl], TNF-α [pg/dl]].
  Other local secondary outcomes: Plaque index (PI), bleeding on probing (BOP), presence of periodontal pockets (PPP), clinical attachment loss (CAL).
  Other secondary systemic outcomes: systolic blood pressure (SBP), diastolic blood pressure (DBP), mean arterial pressure, glucose levels, HDL, low-density lipoprotein (LDL) cholesterol, TG, body mass index (BMI), fibrinogen, central obesity or abdominal circumference.
T: RCTs

Articles were required to be published in English, Spanish or Portuguese, and to appear in journals indexed in the consulted databases. Exclusion criteria encompassed systematic reviews, observational studies with or without comparison groups, editorials, letters to the editor, congress abstracts, and case reports and series.

Different definitions for metabolic syndrome were considered including those of the International Diabetes Federation (IDF) (8), World Health Organization (WHO) (9), National Cholesterol Education Program (NCEP), Adult Treatment Panel III (ATP III) (10) and European Group for the Study of Insulin Resistance (11).

To define periodontal disease, definitions from the American Dental Association (12), the American Academy of Periodontology (13) and the WHO (14) were considered.

### 2.2 Sources of information and search strategies

Searches were conducted in PubMed, EMBASE, CINAHL (Cumulative Index to Nursing and Allied Health Literature), LILACS (Latin American and Caribbean Health Sciences Literature), Scopus, Web of Science (WOS) Core Collection, Dentistry & Oral Science Source, and Cochrane Central databases on June 20, 2023. No language, country of origin or temporal restrictions were applied at the time of the searches. The search strategies used in the different databases are shown in Supplementary Material 1.

### 2.3 Study selection and data extraction

**Figure 1:**
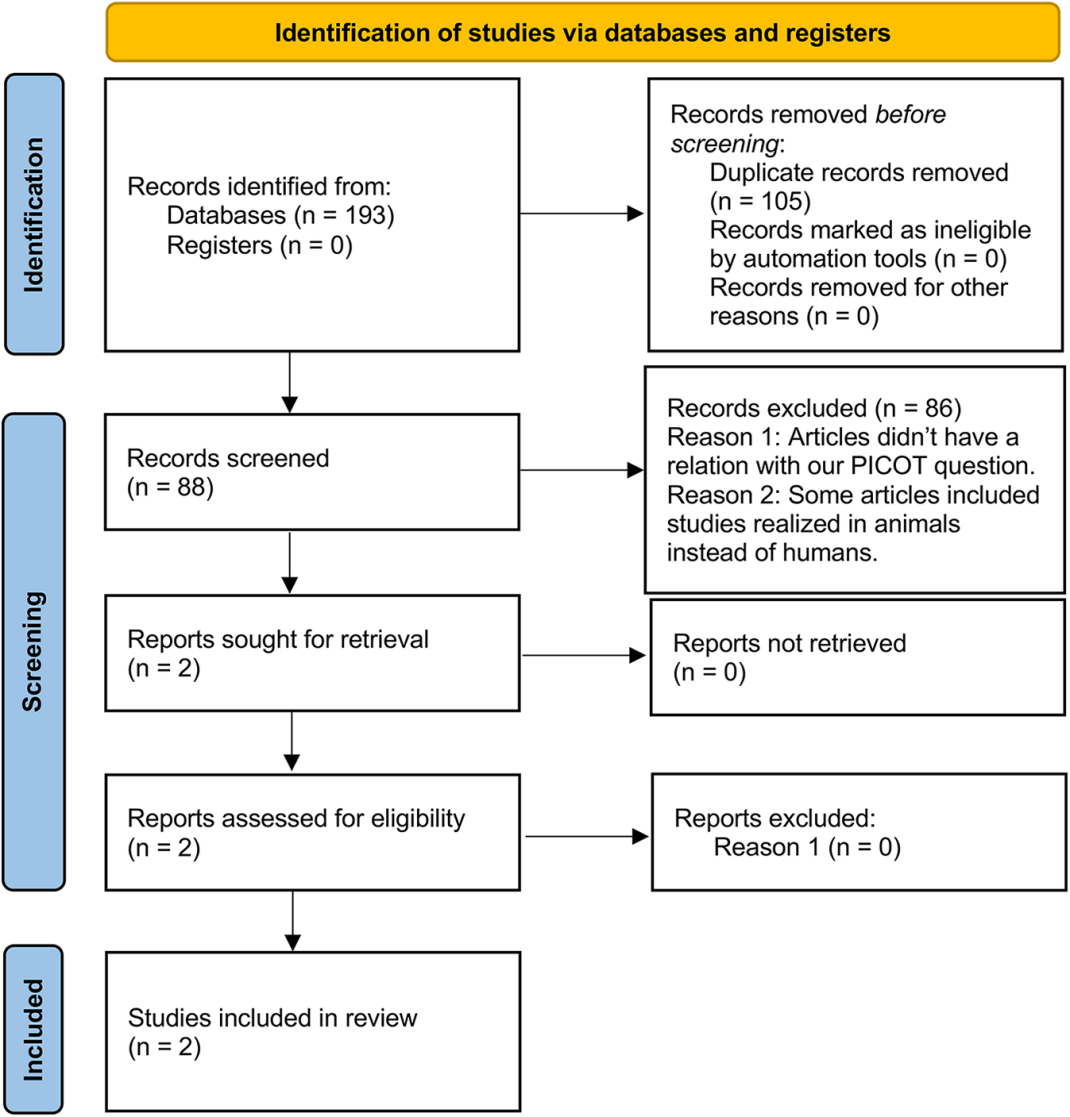
Flowchart of the study selection process. The diagram shows the number of studies identified, evaluated, and selected for inclusion in the systematic review. Initially, 193 studies were identified through database searches and other sources. After removing duplicates, 88 studies were screened by title and abstract, of which 86 were deemed irrelevant. Reasons for exclusion included lack of relevance to our PICOT question and studies conducted on animals instead of humans. Two reports were sought for retrieval, and both were assessed for eligibility. Finally, 2 studies met the inclusion criteria and were included in the review.

The search strategy was developed by the research team in collaboration with an expert medical research librarian (DC). For the elimination of duplicates, the methodology proposed by Bramer et al. (15) was followed. Subsequently, 88 references were obtained and uploaded to the Rayyan web tool (16), and two authors (MC and AR) excluded 86 articles by blind and independent evaluation of their titles and abstracts, leaving only 2 articles selected for full-text review, which preliminarily met the selection criteria (Figure 1). In both phases, discrepancies were resolved by discussion between the two reviewers (MC and AR), and, if the discrepancy persisted, a third author (DA) was consulted.

A data extraction table was made in Excel to collect information from scientific studies. Two authors, MC and AR, entered the following data: article code, author, year of publication, journal name, country, type of study, number of participants, mean age, proportion of patients by sex, intervention and control details, and follow-up time. In addition, baseline and follow-up values of inflammatory markers (CRP mg/L, interleukins pg/dl, TNF pg/dl), as well as estimators of PI, BOP, PPP, CAL, blood pressure, glucose levels, HDL cholesterol, LDL cholesterol, TG, BMI, and fibrinogen were recorded.

**Figure 2:**
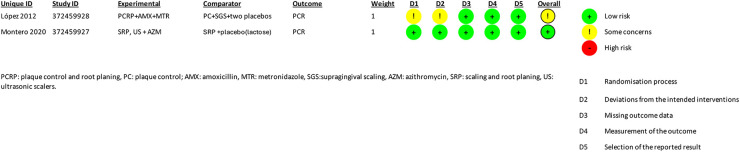
Summary of study characteristics and risk of bias assessments. This table presents the details of the studies included in the review, including study IDs, experimental and comparator groups, outcomes, and risk assessments across various domains. The outcome measured in both studies was CPR (C-reactive protein). López 2012 was assessed as low risk overall, while Montero 2020 had some concerns overall and high risk in specific domains..

**Figure 3:**
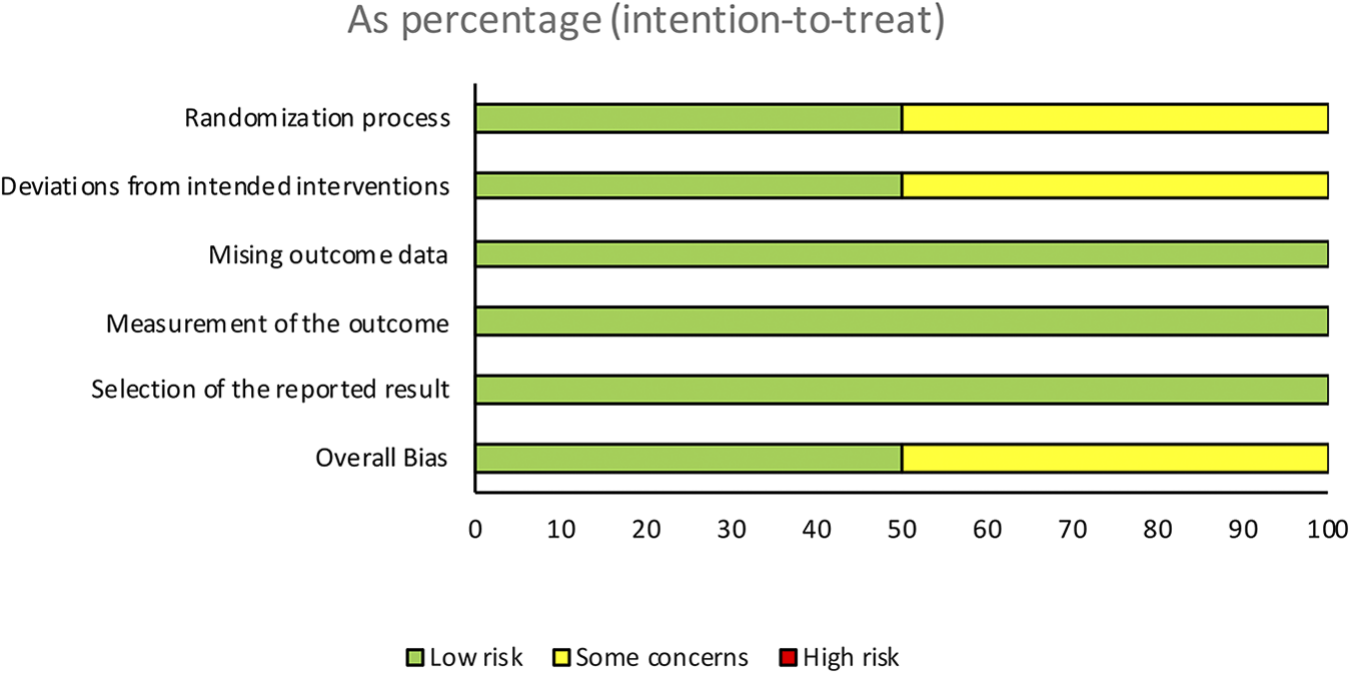
Risk of bias assessment across different domains. This figure illustrates the risk of bias assessments for the included studies across various domains. The domains evaluated include the randomization process, deviations from intended interventions, missing outcome data, measurement of the outcome, and selection of the reported result. The overall bias is also presented. The risk levels are categorized as low risk, some concerns, and high risk, and are displayed as percentages based on an intention-to-treat analysis.

**Figure 4:**
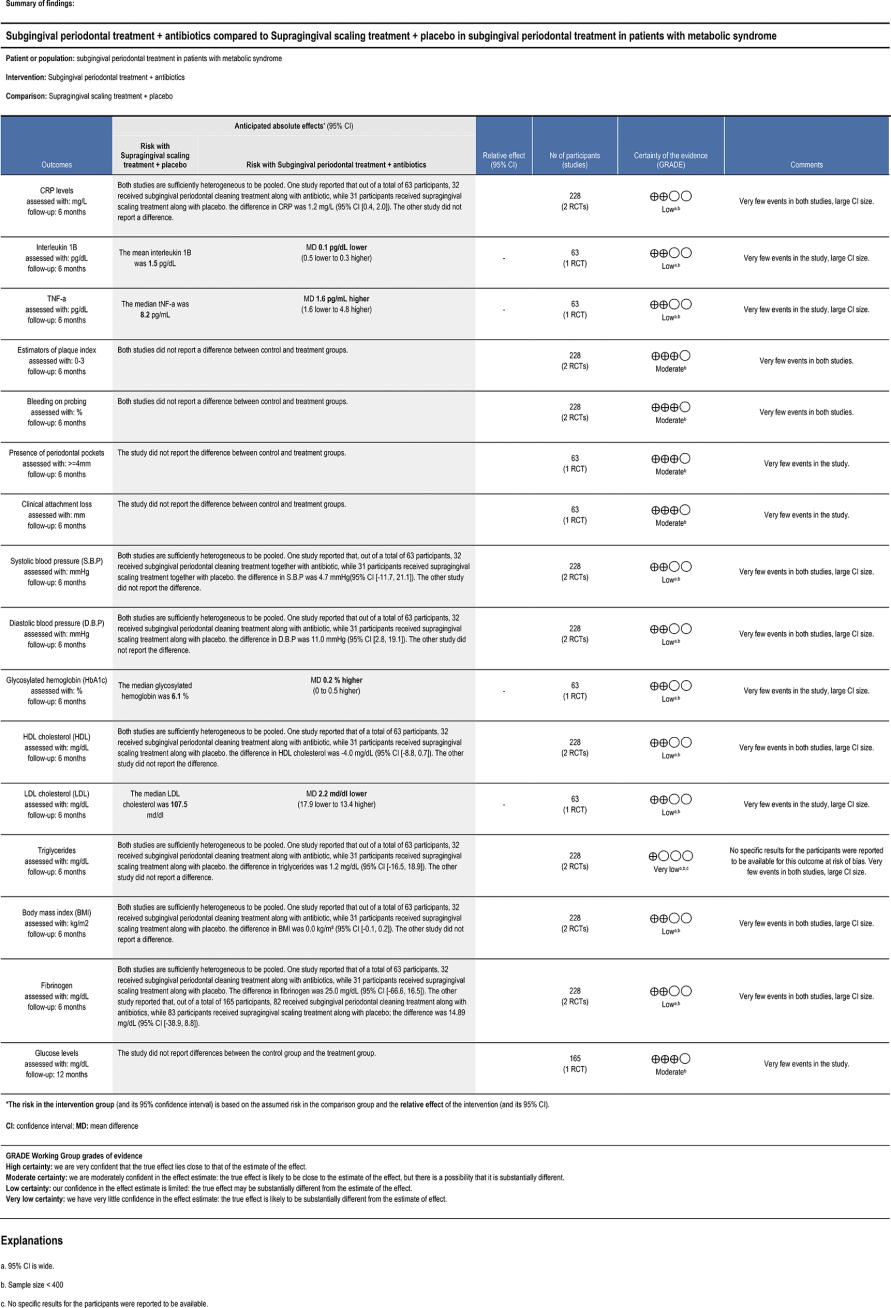
Summary of findings.

The quality assessment of the studies was performed independently and blinded by two investigators using version 2 of the Cochrane Revised Risk of Bias Assessment Tool for Randomized Trials (RoB 2) (17) (Figures 2, 3). The certainty of evidence for each outcome was assessed according to The Grading of Recommendations Assessment, Development and Evaluation (GRADE) guidelines (18) (Figure 4).

## 3 Results

### 3.1 Study selection

The search strategy led to the identification of 88 study records. Two of the records were included in the title and abstract selection, which were also included in the full-text evaluation, after assessing their eligibility using the pre-established selection criteria [López et al. (5); Montero et al. (6)].

### 3.2 Characteristics of the included studies

The study presented by Montero et al. is defined as a double-blind, parallel-arm RCT. Participants were randomized to receive either IPT, which included scaling and root planing (subgingival treatment) along with daily administration of azithromycin 500 mg for 3 days, or minimal periodontal treatment, which was comprised of supragingival professional mechanical removal of dental plaque plus placebo administration. This study was conducted over a 6-year period, from November 2012 to June 2018, with a 6-month follow-up. The research was conducted in Fuenlabrada, Madrid, Spain. Initially, participants were evaluated at the beginning of the study, and then underwent follow-ups at 3 and 6 months after the intervention. These follow-ups included clinical periodontal measurements, such as PPP, clinical attachment level and BOP, as well as PI and gingival index estimators. In addition, blood tests were performed to evaluate inflammatory markers, including serum levels of high-sensitivity CRP (hs-CRP), α-1 antitrypsin, fibrinogen and other markers, such as interleukins (IL-1β, IL-6, IL-8) and TNF-α. Metabolic parameters, such as glycosylated hemoglobin (HbA1c), fasting glucose, lipids and blood pressure, were also evaluated.

On the other hand, the study by López et al. was a double-blind, parallel-arm RCT, in which 2 treatment groups were also established. One group of patients underwent plaque control, scaling and root planing, together with systemic antibiotic treatment of amoxicillin 500 mg and metronidazole 250 mg, 3 times a day for 7 days, 1 week before starting root planing, while the other group received instructions for plaque control, supragingival scaling and two placebos instead of the specific antibiotics. This study was conducted in Santiago, Chile, and followed the participants for a period of one year. During this time, follow-up visits were made at 3, 6, 9 and 12 months after the experimental therapy. At these visits, periodontal clinical parameters, such as PI and BOP estimators, were recorded. In addition, medical evaluations were performed including measurements of cholesterol, glucose, BMI, blood pressure, CRP and blood fibrinogen.

A total of 228 participants, aged between 35 and 65 years, were included in the two selected studies. Both studies evaluated CRP levels at 3 and 6 months, while only one extended the evaluation to 9 and 12 months (5). In the case of Montero et al.*,* the primary outcomes were hs-CRP, inflammatory markers, such as α-1 antitrypsin, fibrinogen, IL-1β, IL-6, IL-8, TNF-α, and as secondary outcomes metabolic parameters including HbA1c, fasting glucose and lipid profile (total cholesterol, HDL cholesterol, LDL cholesterol, TG), BMI and blood pressure, as well as periodontal clinical parameters, such as PI, BOP, BP, and clinical attachment level. In the study by López et al., only CRP was considered as the primary outcome. Secondary outcomes included fibrinogen, as well as periodontal parameters, such as PI and BOP, metabolic parameters including fasting glucose, lipid profile (HDL cholesterol, TG), BMI and blood pressure.

### 3.3 Assessment of risk of bias of the results

The summary of the risk of bias is illustrated in Figures 2, 3. Regarding bias for the primary outcome of CRP, the study by Montero et al. indicated a low risk of bias, whereas the study by Lopez et al. was rated as unclear.

Regarding the randomization process, the study by López et al. was rated with unclear risk of bias, because it used a minimization method to assign the participants to the treatment groups due to differences in baseline characteristics, with the aim of reducing possible bias in the assignment. Montero's study was rated as low risk of bias.

In relation to the intended interventions, the study by Montero et al. had a low risk of bias. However, the study by López et al. was unclear, as they excluded data from patients with recurrent systemic infections due to the high and distorted CRP values associated with acute infections. This exclusion was considered necessary according to the authors, to avoid compromising the study objectives and affecting the intention-to-treat analysis. It is relevant to note that the excluded participants represented less than 5% of the total.

Regarding missing outcome data, in both studies, a low risk of bias was observed due to the availability of data for most participants. However, in the Lopez et al. study, data from patients with recurrent infections were excluded, with data being available for more than 95% of participants.

In terms of outcome measurement, both studies had a low risk of bias due to double blinding of the studies and appropriate methods to measure the results.

Regarding the selection of reported CRP outcomes, both studies demonstrated a low risk of bias. This was because they used pre-specified data analyses for this outcome before the actual results were known. Furthermore, in the Lopez et al. study, all CRP results were reported both in their original form and after logarithmization of the result.

### 3.4 Evaluation of the certainty of the results

The results are summarized in Figure 4.

In this systematic review, the studies by López et al. and Montero et al. were found to have a very low to moderate certainty. In the analysis, all variables had a level of certainty ranging from low to moderate, except for TG. The decrease in certainty was attributed to two common factors: first, a sample size of less than 400 patients affects precision. In addition, the presence of wide confidence intervals also contributes to a further reduction in the level of precision in these outcomes (CRP, IL-1β, TNF-α, SBP, DBP, HbA1c, TG, BMI, fibrinogen, LDL and HDL) (19). On the other hand, in relation to the TG variable, it was the only variable with a very low certainty. This is due to the risk of bias, which turned out to be serious due to the missing data in that result, and when considering the two factors mentioned above, the certainty decreased considerably.

### 3.5 Individual results of the studies included

#### 3.5.1 Primary outcomes

##### 3.5.1.1 CRP

Only one of the studies reported significant differences in favor of the treatment group. Montero et al. found significant differences in favor of the treatment group at 3 months [2.7 mg/L ± standard error (SE): 0.4 vs. 3.9 mg/L ± SE: 0.6; *p* = 0.001] and at 6 months (2.9 mg/L ± SE: 0.4 vs. 4.0 mg/L ± SE: 0.8; *p* = 0.004). On the other hand, Lopez et al. reported no significant differences at 3 months (4.25 mg/L vs. 4.40 mg/L), 6 months (4.58 mg/L vs. 3.57 mg/L), 9 months (4.2 mg/L vs. 4.31 mg/L) and at 12 months (3.62 mg/L vs. 2.91 mg/L) (Table 1).

**Table 1:** Subgingival periodontal treatment + antibiotics vs. supragingival cleaning treatment + placebo in patients with metabolic syndrome and periodontal disease.

Article	Procedure		Primary outcomes		Local secondary outcomes		Systemic secondary outcomes	
	Treatment group	Control group	Treatment group	Control Group	Treatment group	Control Group	Treatment group	Control Group
López et al. (5)	PCRP + AMX + MTR	PC + SGS + two placebo	CRP (mg/L) (mean ± SD)<>		Estimators of plaque index (%) (mean ± SD)		Systolic Blood pressure (mmHg) (mean ± SD)<>	
			BL: 4.43 ± 3.05	BL: 4.39 ± 3.17	BL: 87.96 ± 13.89	BL: 85.15 ± 16.62	BL: 147 ± 6.8∼	BL: 149 ± 6.9∼
			3 months: 4.25^^,^¶	3 months: 4.40^^,^¶	3 months: **53.26**^^,^¶	3 months: **56.20**^^,^¶	3 months: 143.13^^,^¶	3 months: 144.27^^,^¶
			6 months: 4.58^^,^¶	6 months: 3.57^^,^¶	6 months: **51.22**^^,^¶	6 months: **55.29**^^,^¶	6 months: 144.08^^,^¶	6 months: 146.16^^,^¶
			9 months: **4.2** ¶	9 months: **4.31**¶	9 months: **51.22**^^,^¶	9 months: **55.07**^^,^¶	9 months: 147.11^^,^¶	9 months: 145.97^^,^¶
			12 months: **3.62**¶	12 months: **2.91**¶	12 months: 48.03*^,^^^,^¶	12 months: 56.20*^,^^^,^¶	12 months: 146.16^^,^¶	12 months: 145.02^^,^¶
			Log CRP (mg/L)<>		Bleeding on probing (% of sites) (mean ± SD)		Diastolic Blood pressure (mmHg) (mean ± SD)<>	
			BL: 1.20¶,∼	BL: 1.20¶,∼	BL: 51.54 ± 17.13	BL: 51.22 ± 16.86	BL: 97 ± 4.1∼	BL: 98 ± 3.8∼
			3 months: 1.06^^,^¶	3 months: 1.24^^,^¶	3 months: 22.50*^,^^^,^¶	3 months: 36.61*^,^^^,^¶	3 months: 95.92^^,^¶	3 months: 94.98^^,^¶
			6 months: 1.17^^,^¶	6 months: 1.00^^,^¶	6 months: 21.96*^,^^^,^¶	6 months: 35.89*^,^^^,^¶	6 months: 93.08^^,^¶	6 months: 94.22^^,^¶
			9 months: 1.06^^,^¶	9 months: 0.82^^,^¶	9 months: 22.86*^,^^^,^¶	9 months: 35.18*^,^^^,^¶	9 months: 94.98^^,^¶	9 months: 96.11^^,^¶
			12 months: 0.79^^,^¶	12 months: 0.43^^,^¶	12 months: 22.32*^,^^^,^¶	12 months: 35.36*^,^^^,^¶	12 months: 94.03^^,^¶	12 months: 95.92^^,^¶
							Glucose levels (mg/dl) (mean ± SD)<>	
							BL: 112.24 ± 45.16∼	BL: 106.60 ± 43.66∼
							3 months: 110.58^^,^¶	3 months: 110.58^^,^¶
							6 months: 115.57^^,^¶	6 months: 110.58^^,^¶
							9 months: 110.99^^,^¶	9 months: 112.24^^,^¶
							12 months: 112.24^^,^¶	12 months: 115.57^^,^¶
							HDL cholesterol (mg/dl) (mean ± SD)<>	
							BL: 50.00 ± 12.87∼	BL: 50.18 ± 14.63∼
							3 months: 50.96^^,^¶	3 months: 51.86^^,^¶
							6 months: 50.12^^,^¶	6 months: 50.57^^,^¶
							9 months: 48.82^^,^¶	9 months: 50.62^^,^¶
							12 months: 48.20^^,^¶	12 months: 50.68^^,^¶
							Triglycerides (mg/dl) (mean ± SD)<>	
							BL: 174.90 ± 101.13∼	BL: 158.80 ± 118.23∼
							3 months: 173.55^^,^¶	3 months: 167.98^^,^¶
							6 months: 182.83^^,^¶	6 months: 170.77^^,^¶
							9 months: 185.61^^,^¶	9 months: 173.55^^,^¶
							12 months: 185.61^^,^¶	12 months: 174.48^^,^¶
							BMI (kg/m^2^) (mean ± SD)<>	
							BL: 29.96 ± 3.89∼	BL: 30.39 ± 4.26∼
							3 months: 29.93^^,^¶	3 months: 30.09^^,^¶
							6 months: 30.51^^,^¶	6 months: 29.96^^,^¶
							9 months: 30.60^^,^¶	9 months: 29.86^^,^¶
							12 months: 30.54^^,^¶	12 months: 29.97^^,^¶
							Fibrinogen (mg/dl) (mean ± SD)<>	
							BL: 378 ± 102∼	BL: 337 ± 115∼
							3 meses: 360.83^^,^¶	3 meses: 348.16^^,^¶
							6 meses: 338.25¶	6 meses: 350.69^^,^¶
							9 meses: 358.06^^,^¶	9 meses: 360.83^^,^¶
							12 meses: 352.53¶	12 meses: 345.62^^,^¶
Montero et al. (6)	SRP, US + AZM	SRP + placebo (LACTOSE)	CRP levels mg/L [mean (SE)]		Estimators of plaque index (0–3) (mean ± SD)		Systolic Blood pressure, mmHg [mean (SE)]	
			BL: 3.9 ± 2.9∼	BL: 3.9 ± 3.4∼	BL: 1.8 ± 0.4	BL: 1.9 ± 0.5	BL: 148.1 ± 21.5	BL: 138.6 ± 18.3∼
			3 months: 2.7 ± 0.4*	3 months: 3.9 ± 0.6*	3 months: **0.8** ± 0.3*	3 months: **1.3** ± 0.7*	3 months: **136.4** ± 3.0*	3 months: 139.4 ± 2.7*
			6 months: 2.9 ± 0.4*	6 months: 4.0 ± 0.8*	6 months: **0.8** ± 0.3*	6 months: **1.2** ± 0.6*	6 months: 136.4 ± 2.8	6 months: 144.4 ± 4.1
			Interleukins-1B pg/dl [mean (SE)]		Bleeding on probing (%)(mean ± SD)		Diastolic Blood pressure, mmHg[mean (SE)]	
			BL: 1.5 ± 0.9∼	BL: 1.9 ± 1.2∼	BL: 59.8% ± 20	BL: 67.8% 20	BL: 91.3 ± 18.2∼	BL: 84.1 ± 11.2∼
			3 months: 0.9 ± 0.1*	3 months: 2.3 ± 0.5*	3 months: **24.9%** ± 17*	3 months: **48.4%** ± 21*	3 months: 84.8 ± 3.8*	3 months: 89.6 ± 5.4*
			6 months: 1.5 ± 0.2	6 months: 1.5 ± 0.2	6 months: **20.5%** ± 11*	6 months: 51.6% ± 18*	6 months: 81.8 ± 2.8*	6 months: 86.9 ± 1.8*
			TNF-α pg/dl [mean (SE)]		Presence of periodontal pockets >4 mm (mean ± SD)		HbA1c, %, [mean (SE)]	
			BL: 7.9 ± 6.2	BL: 8.7 ± 8.6∼	BL: 55.3% ± 22	BL: 59.7% ± 19	BL: 6.3 ± 1.2	BL: 6.0 ± 1.0∼
			3 months: **6.4** ± 0.8*	3 months: 10.0 ± 2.3*	3 months: **13.9%** ± 12*	3 months: **41.2%** ± 23*	3 months: **5.9** ± 0.1*	3 months: 6.1 ± 0.2*
			6 months: **6.3** ± **0.8**	6 months: 8.2 ± 1.4	6 months: **11.2%** ± 10*	6 months: **43.9%** ± 21*	6 months: **6.0** ± **0.1**	6 months: 6.1 ± 0.2
					Clinical attachment loss/leves, mm (mean ± SD)		HDL cholesterol, mg/dl[mean (SE)]	
					BL: 4.9 ± 1.0	BL: 5.2 ± 1.3∼	BL: 46.1 ± 13.3∼	BL: 46.9 ± 12.4∼
					3 months: **3.9** ± 1.0*	3 months: 4.8 ± 1.4*	3 months: 46.2 ± 3.8	3 months: 47.1 ± 3.1
					6 months: **3.8** ± 0.9*	6 months: 4.9 ± 1.3*	6 months: 47.2 ± 2.7	6 months: 48.4 ± 2.7
							LDL cholesterol, mg/dl[mean (SE)]	
							BL: 114.3 ± 34.7∼	BL: 105.7 ± 44.9∼
							3 months: 109.6 ± 8.5	3 months: 103.5 ± 7.0
							6 months: 107.6 ± 6.6	6 months: 107.5 ± 8.3
							Triglycerides, mg/dl[mean (SE)]	
							BL: 129.5 ± 52.3∼	BL: 136.6 ± 42.5∼
							3 months: 136.5 ± 9.7	3 months: 155.4 ± 17.5
							6 months: 125.6 ± 9.7	6 months: 131.7 ± 8.3
							BMI, kg/m^2^ [mean (SE)]	
							BL: 39.1 ± 5.6∼	BL: 38.0 ± 4.7∼
							3 months: 39.1 ± 1.6	3 months: 38.0 ± 1.6
							6 months: 39.2 ± 1.6	6 months: 38.0 ± 1.6
							Fibrinogen [mean (SE)]	
							BL: 419.7 ± 108.7∼	BL: 398.5 ± 89.1∼
							3 months: 421.8 ± 20.4	3 months: 398.3 ± 17.9
							6 months: 419.6 ± 21.8	6 months: 400.5 ± 16.1

Abbreviations: PCRP, plaque control and root planing; PC, plaque control; AMX, amoxicillin; MTR, metronidazole; SGS, supragingival scaling; AZM, azithromycin; SRP, scaling and root planing; US, ultrasonic scalers; NSPT, non-surgical periodontal therapy; BL, Baseline; SE, Standar Error; SD, Standar Desviation; BMI, body mass index; TNF-α, tumor necrosis factor-alpha; CRP, C-reactive protein; HbA1c, glycosylated hemoglobin; HDL, high-density lipoprotein; LDL, low-density lipoprotein; CPT.

Numbers in bold represent differences within treatment or control groups compared to baseline, where the significance level is ≤0.05.

Results of the evaluated outcomes are expressed in means, or means and standard deviations unless otherwise specified.

* Asterisks represent differences between groups at different points of follow-up.

^ Represents that *p* values are not reported.

∼Represents that the changes were not significant.

<> The study does not report whether there were any statistically significant differences between groups.

^¶^ Represents that standard deviations are not reported.

##### 3.5.1.2 IL-1β

Only the study by Montero et al. reported IL-1β values. describing significant differences in favor of the treatment group at 3 months (0.9 pg/dl ± SE: 0.1 vs. 2.3 pg/dl ± SE: 0.5; *p* = 0.046) but not at 6 months (1.5 pg/dl ± SE: 0.2 vs. 1.5 pg/dl ± SE: 0.2; *p* = 0.601) (Table 1).

##### 3.5.1.3 TNF-α

Only one of the studies reported TNF-α values, with Montero et al. finding significant differences in favor of the treatment group at 3 months (6.4 pg/dl ± SE: 0.8 vs. 10.0 pg/dl ± SE: 2.3; *p* = 0.037) but not at 6 months (6.3 pg/dl ± SE: 0.8 vs. 8.2 pg/dl ± SE: 1.4; *p* = 0.333) (Table 1).

#### 3.5.2 Local secondary outcomes

##### 3.5.2.1 PI

Both studies showed reductions in the percentage of PI in the treatment compared to the control group. According to Montero et al.*,* there were significant differences in both groups in favor of the treatment group at 3 months [0.8% ± 0.3 standard deviation [SD] vs. 1.3% ± 0.7(SD); *p* = 0.002] and at 6 months [0.8% ± 0.3(SD) vs. 1.2% ± 0.6(SD); *p* = 0.013]. On the other hand, Lopez et al. reported no significant differences at 3 months (53.26% vs. 56.20%; *p* > 0.05), 6 months (51.22% vs. 55.29%; *p* > 0.05), 9 months (51.22% vs. 55.07%; *p* > 0.05) or at 12 months (48.03% vs. 56.20%; *p* < 0.05) (Table 1).

##### 3.5.2.2 BOP

The findings of both studies suggest significant decreases in the percentage of BOP in favor of the treatment group, mainly at 6 months of follow-up. According to Montero et al., there were significant differences in favor of the treatment group at 3 months [24.9% ± 17(SD) vs. 48.4.8% ± 21(SD); *p* < 0.001] and at 6 months [20.5% ± 11(SD) vs. 51.6% ± 18(SD); *p* < 0.001]. On the other hand, Lopez et al. reported significant differences in favor of the treatment group at 3 months (22.50% vs. 36.61%; *p* ≤ 0.05), 6 months (21.96% vs. 35.89%; *p* ≤ 0.05), 9 months (22.86% vs. 35.18%; *p* ≤ 0.05) and at 12 months (22.32% vs. 35.36%; *p* ≤ 0.05) (Table 1).

##### 3.5.2.3 PPP

Only one of the studies reported PPP values. Montero et al. described significant differences in favor of the treatment group at 3 months [13.9% ± 12(SD) vs. 41.2% ± 23(SD); *p* < 0.001] and at 6 months [11.2% ± 10(SD) vs. 43.9% ± 21(SD); *p* < 0.001] (Table 1).

##### 3.5.2.4 CAL

Only the study by Montero et al. reported CAL values, finding significant differences in favor of the treatment group at 3 months [3.9 mm ± 1.0 (SD) vs. 4.8 mm ± 1.4 (SD); *p* = 0.010] and at 6 months [3.8 mm ± 0.9 (SD) vs. 4.9 mm ± 1.3 (SD); *p* < 0.001] (Table 1).

#### 3.5.3 Systemic secondary outcomes

##### 3.5.3.1 SBP

Only one of the studies reported significant differences in SBP values at 3 months. According to Montero et al. there were significant differences in favor of the treatment group at 3 months (136.4 mmHg ± SE: 3.0 vs. 139.4 ± SE: 2.7; *p* = 0.008) but not at 6 months (136.4 mmHg ± SE: 2.8 vs. 144.4 mmHg ± SE: 4.1; *p* = 0.574). On the other hand, López et al. reported no significant differences at 3 months (143.13 mmHg vs. 144.2 mmHg; *p* > 0.05), 6 months (144.08 mmHg vs. 146.16 mmHg; *p* > 0.05), 9 months (147.11 mmHg vs. 145.97 mmHg) or at 12 months (146.16 mmHg vs. 145.02 mmHg) (Table 1).

##### 3.5.3.2 DBP

A decrease in DBP was observed in the treatment groups. Montero et al. reported significant differences in favor of the treatment group at 3 months (84.8 mmHg ± SE: 3.8 vs. 89.6 mmHg ± SE: 5.4; *p* = 0.019) and at 6 months (81.8 mmHg ± SE: 2.8 vs. 86.9 mmHg + SE: 1.8; *p* = 0.009). On the other hand, López et al. found no significant differences at 3 months (95.92 mmHg vs. 94.98 mmHg; *p* > 0.05), 6 months (93.08 mmHg vs. 94.22 mmHg; *p* > 0.05), 9 months (94.98 mmHg vs. 96.11 mmHg; *p* > 0.05) or at 12 months (94.03 mmHg vs. 95.92 mmHg; *p* > 0.05) (Table 1).

#### 3.5.4 Mean arterial pressure

No outcomes were identified for this outcome.

##### 3.5.4.1 HbA1c

Only one of the studies reported HbA1c values. Montero et al. described significant differences in favor of the treatment group only at 3 months (5.9% ± SE: 0.1 vs. 6.1% ± SE: 0.2; *p* = 0.013) but not at 6 months (6.0% ± SE: 0.1 vs. 6.1% ± SE: 0.2; *p* = 0.110) (Table 1).

##### 3.5.4.2 Glucose levels

Only the study by Lopez et al. (5) reported glucose values and found no significant differences at 3 months (110.58 mg/dl vs. 110.58 mg/dl; *p* > 0.05), 6 months (115.57 mg/dl vs. 110.58 mg/dl; *p* > 0.05), 9 months (110.99 mg/dl vs. 112.24 mg/dl; *p* > 0.05).05), 9 months (110.99 mg/dl vs. 112.24 mg/dl; *p* > 0.05) or at 12 months (112.14 mg/dl vs. 115.57 mg/dl; *p* > 0.05) (Table 1).

##### 3.5.4.3 HDL cholesterol

No significant differences were found between the treatment and control groups. Montero et al. reported no significant differences at 3 months (46.2 mg/dl ± SE: 3.8 vs. 47.1 mg/dl + SE: 3.1; *p* = 0.858) or at 6 months (47.2 mg/dl ± SE: 2.7 vs. 48.4 mg/dl ± SE: 2.7; *p* = 0.097). López et al. also reported no significant differences at 3 months (50.96 mg/L vs. 51.86 mg/L; *p* > 0.05), 6 months (50.12 mg/L vs. 50.57 mg/L; *p* > 0.05), 9 months (48.82 mg/L vs. 50.62 mg/L; *p* > 0.05) or at 12 months (48.20 mg/L vs. 50.68 mg/L; *p* > 0.05) (Table 1).

##### 3.5.4.4 LDL cholesterol

Only one of the studies reported LDL cholesterol values. Montero et al. found no significant differences at either 3 months (109.6 mg/dl ± SE: 8.5 vs. 103.5 mg/dl ± SE: 7.0; *p* = 0.327), or at 6 months (107.6 mg/dl + SE: 6.6 vs. 107.5 mg/dl + SE: 8.3; *p* = 0.779) (Table 1).

##### 3.5.4.5 TG

In relation to TG values, the study by Montero et al. reported no significant differences at 3 months (136.5 mg/dl + SE: 9.7 vs. 155.4 mg/dl + SE: 17.5; *p* = 0.984) or at 6 months (125.6 mg/dl + SE: 9.7 vs. 131.7 mg/dl + SE: 8.3; *p* = 0.895). López et al. also reported no significant differences at 3 months (173.55 mg/L vs. 167.98 mg/L; *p* > 0.05) 6 months (182.83 mg/L vs. 170.77 mg/L; *p* > 0.05) 9 months (185.61 mg/L vs. 173.55 mg/L; *p* > 0.05) or at 12 months (185.61 mg/L vs. 174.48 mg/L; *p* > 0.05) (Table 1).

##### 3.5.4.6 BMI

No significant changes were reported in either study. The BMI values of the treatment and control groups were maintained at 6 and 12 months. Montero et al. reported no significant differences at 3 months (39.1 kg/m^2^ + SE: 1.6 vs. 38.0 kg/m^2^ + SE: 1.6; *p* = 0.503) or at 6 months (39.2 kg/m^2^ + SE: 1.6 vs. 38. 0 kg/m^2^ + SE: 1.6; *p* = 0.611). In addition, López et al. reported no significant differences at 3 months (29.93 kg/m^2^ vs. 30.09 kg/m^2^; *p* > 0.05), 6 months (30.51 kg/m^2^ vs. 29.96 kg/m^2^; *p* > 0.05), 9 months (30.60 kg/m^2^ vs. 29.86 kg/m^2^; *p* > 0.05) or at 12 months (30.54 kg/m^2^ vs. 29.97 kg/m^2^; *p* > 0.05) (Table 1).

##### 3.5.4.7 Fibrinogen

No significant differences in favor of any of the treatment groups were observed in either study. According to Montero et al.*,* there were no significant differences at 3 months (421.8 mg/dl + SE: 20.4 vs. 398.3 mg/dl + SE: 17.9; *p* = 0.144) or at 6 months (419.6 mg/dl + SE: 21.8 vs. 400.5 mg/dl + SE: 16.1; *p* = 0.238). López et al. also reported no significant differences at 3 months (360.83 mg/dl vs. 348.16 mg/dl; *p* > 0.05), 6 months (338.25 mg/dl vs. 350.69 mg/dl; *p* > 0.05), 9 months (358.06 mg/dl vs. 360.83 mg/dl; *p* > 0.05) or at 12 months (352.53 mg/dl vs. 345.62 mg/dl; *p* > 0.05) (Table 1).

## 4 Discussion

The aim of the present study was to synthesize the evidence available on the impact of subgingival periodontal treatment + antibiotic therapy on the reduction of systemic markers of inflammation [CRP (mg/L), and interleukins (pg/dl), TNF-α (pg/dl)] in patients with metabolic syndrome and periodontal disease, compared with supragingival periodontal treatment + placebo. After searching and selecting the evidence, two RCTs were included (5, 6). The certainty of evidence of the two studies ranged from very low to moderate due to several factors. One of the main factors was imprecision, mainly due to the small sample size and wide confidence intervals for some outcomes (19).

According to the results of Montero et al.*,* the combination of periodontal therapy with antibiotic therapy can have a positive effect on systemic inflammation (reflected in CRP, TNF-α, IL-1β levels) in patients with metabolic syndrome and periodontal disease. This study showed a low risk of bias and low-moderate quality of certainty of evidence for the mentioned outcomes (6). CRP is a marker of acute-phase inflammation that was recently postulated as a marker of atherogenesis and as a predictor of cardiovascular events, because the proinflammatory state of the arteries can cause fat particles and LDL cholesterol to deposit on the arterial walls, forming plaques (20, 21, 22). These plaques can harden over time and narrow the arteries, impeding blood flow and increasing the risk of cardiovascular events (22). Chronic periodontal disease can trigger a systemic inflammatory response in the body, mediated by proinflammatory factors such as TNF-α, IL-1 and IL-6, which, in turn, stimulate hepatic production of CRP (23). Treatment of periodontal infection with antibiotics, removing plaque and dental calculus can reduce local inflammation and, thus, decrease systemic inflammation, which is reflected by lower CRP levels (24). In this sense, these findings suggest that the concomitant use of subgingival periodontal treatment and antibiotic therapy may be beneficial in the clinical management of these patients.

According to the study by Lopez et al. subgingival periodontal treatment has a significant impact on local inflammation (5). By removing plaque and bacterial calculus from periodontal pockets, this treatment can reduce PI, BOP, PBP and ICP levels. Moreover, the effect of this treatment is further enhanced when combined with the use of antibiotics. Therefore, it is essential to consider these strategies in the management of patients with metabolic syndrome and periodontal disease (24–26). On the other hand, the study by Montero et al. mentions that effective periodontal treatment is one that combines subgingival treatment with antibiotics, leading to significant reductions in biomarkers of inflammation, such as CRP and TNF-α (6). In addition, improvement in vascular function and metabolic control was observed compared to the control group in which CRP, SBP and HbA1c decreased following supragingival professional mechanical removal of dental plaque along with placebo. The reduction in bacterial load also decreases anaerobic bacterial counts and *Porphyromonas gingivalis*, leading to decreased CRP values and, thus, a reduction in cardiovascular events (6, 27).

With respect to IL-1β, significant differences between groups were observed only at 3 months in favor of the treatment group. Variations in IL-1β levels were minimal when comparing the initial values with the values at the different follow-ups. This could be due to the short period of antibiotic use in the treatment group. According to Borges et al. longer exposure periods up to 14 days of antibiotics may be required to eradicate microorganisms residing in the highly organized structure of the subgingival biofilm (28). However, they were able to demonstrate significant results in secondary outcomes.

In relation to HbA1c, significant reductions associated with the treatment group were evidenced. In addition, a reduction in SBP was observed, suggesting that this benefit is not only limited to the improvement of inflammation in the body, but also positively influences vascular function and metabolic control (29, 30). Importantly, periodontal inflammation has been associated with insulin resistance and endothelial dysfunction. One of the key mechanisms involved in this relationship is the chronic low-grade inflammation associated with periodontal disease (24, 31). This inflammation releases inflammatory mediators and cytokines that may contribute to insulin resistance and impaired pancreatic beta-cell function, leading to poorer blood glucose control in patients with type 2 diabetes (32). In addition, periodontal inflammation can induce endothelial dysfunction, increasing the risk of cardiovascular disease, which is a major concern in people with diabetes (1). Periodontal inflammation not only impacts oral health, but also has significant systemic consequences. Therefore, it is crucial that people with diabetes pay special attention to their oral health to mitigate these additional risks.

Taken together, these studies support the idea that effective periodontal treatment can have a positive impact on the overall health of patients with metabolic syndrome and periodontitis by reducing systemic inflammation, improving vascular function and reducing cardiovascular risk. However, the limited availability of RCTs, with small sample size, lack of standardization of interventions and follow-up periods should be taken into consideration.

## 5 Recommendations

The present study allowed the identification of some gaps in knowledge. First, with the available studies it is not possible to know whether the effect observed with respect to markers of systemic inflammation is due to the combination of periodontal treatment + antibiotic or whether it could be attributed exclusively to periodontal treatment; therefore, it would be valuable to conduct RCTs that evaluate only periodontal treatment to observe whether this could improve markers of inflammation. RCTs with larger sample sizes, standardized methods, and longer follow-up times are needed to obtain greater certainty regarding the effect of this intervention and the possibility of comparing other treatment groups, such as supragingival vs. subgingival treatment.

Intensive treatment should be considered in patients with metabolic syndrome who have elevated CRP levels as the main treatment target. Other inflammatory markers, such as IL-6, IL-8, and acute phase reactants of inflammation, should also be taken into account t as they are the first to manifest in the face of clinical improvement or an infectious process (leukocyte count, erythrocyte sedimentation rate and serum amyloid A proteins). New RCTs should also include glucose and TG data, which can be useful to conduct further research to better understand these aspects in relation to the treatment evaluated.

It is crucial for patients with metabolic syndrome to receive regular follow-up by a dental professional to improve their periodontal health and reduce systemic inflammation.

## 6 Limitations

The main limitation of this study was that we found a small number of comparative RCTs on the subject. However, the search was conducted by an expert who ensured an exhaustive search, managing to collect as many eligible studies as possible. On the other hand, it is possible that studies published in languages other than Spanish, Portuguese and English are available, which could have resulted in the loss of relevant evidence to answer the research question. Nevertheless, these three languages were the most widely used in the biomedical publications consulted in the databases. Furthermore, a quantitative synthesis of the evidence (meta-analysis) could not be performed due to the small number or heterogeneity of the studies, since most focused on diabetic or coronary patients, but not necessarily on the group of conditions that make up the metabolic syndrome. Finally, it is important to note that the outcomes assessed (systemic markers of inflammation) were surrogates of other clinical outcomes. These markers would have served mainly for hypothesis generation and, to a lesser extent, for decision making.

## 7 Conclusions

In patients with metabolic syndrome and periodontal disease, this review emphasizes the possibility for subgingival periodontal therapy in conjunction with antibiotics to reduce systemic inflammation. The significance of periodontal health in the treatment of systemic inflammatory diseases is highlighted by these findings, which also imply that intensive periodontal care may help at-risk groups more broadly. Confirming these impacts and assessing the best therapeutic approaches—including the possible advantages of antibiotic-free approaches—will require well-designed trials in the future. Improved comprehension of these processes could eventually result in integrated treatment plans that lower systemic inflammation and enhance metabolic syndrome patients' health.

## Data Availability

The original contributions presented in the study are included in the article/Supplementary Material, further inquiries can be directed to the corresponding author.

## References

[1] Daudt LD Musskopf ML Mendez M Remonti LLR Leitão CB Gross JL Association between metabolic syndrome and periodontitis: a systematic review and meta-analysis. Braz Oral Res. (2018) 32:e35. 10.1590/1807-3107bor-2018.vol32.0035 29846383

[2] Hlushchenko TA Batig VM Borysenko AV Tokar OM Batih IV Vynogradova OM Prevalence and intensity of periodontal disease in individuals with metabolic syndrome. J Med Life. (2020) 13(3):289–92. 10.25122/jml-2020-0073 33072198 PMC7550136

[3] Loos BG. Systemic markers of inflammation in periodontitis. J Periodontol. (2005) 76(11S):2106–15. 10.1902/jop.2005.76.11-S.2106 29539043

[4] Haraszthy VI Zambon JJ Trevisan M Zeid M Genco RJ. Identification of periodontal pathogens in atheromatous plaques. J Periodontol. (2000) 71(10):1554–60. 10.1902/jop.2000.71.10.1554 11063387

[5] López NJ Quintero A Casanova PA Ibieta CI Baelum V López R. Effects of periodontal therapy on systemic markers of inflammation in patients with metabolic syndrome: a controlled clinical trial. J Periodontol. (2012) 83(3):267–78. 10.1902/jop.2011.110227 21749167

[6] Montero E López M Vidal H Martínez M Virto L Marrero J Impact of periodontal therapy on systemic markers of inflammation in patients with metabolic syndrome: a randomized clinical trial. Diabetes Obes Metab. (2020) 22(11):2120–32. 10.1111/dom.14131 32613714

[7] Page MJ McKenzie JE Bossuyt PM Boutron I Hoffmann TC Mulrow CD The PRISMA 2020 statement: an updated guideline for reporting systematic reviews. Br Med J. (2021) 372:1–6. Available online at: https://www.bmj.com/content/372/bmj.n71 10.1136/bmj.n71 PMC8005924 33782057

[8] Alberti SG Zimmet P. Worldwide definition for use in clinical practice. In: IDF Communications, editor. IDF Consensus Worldwide Definition of the Metabolic Syndrome. Brussels: International Diabetes Federation (2006). p. 10–12. Available online at: https://idf.org/media/uploads/2023/05/attachments-30.pdf (Cited November 07, 2022).

[9] Alberti KG Zimmet PZ. Definition, diagnosis and classification of diabetes mellitus and its complications. Part 1: diagnosis and classification of diabetes mellitus provisional report of a WHO consultation. Diabet Med. (1998) 15:539–53. 10.1002/(SICI)1096-9136(199807)15:7<539::AID-DIA668>3.0.CO;2-S 9686693

[10] National Cholesterol Education Program (U.S.). Third Report of the National Cholesterol Education Program (NCEP) expert panel on detection, evaluation, and treatment of high blood cholesterol in adults (adult treatment panel III) final report. Circulation. (2002) 106(25):3143–421. 10.1161/circ.106.25.3143 12485966

[11] Balkau B Charles MA. Comment on the provisional report from the WHO consultation. Diabetic Med. (1999) 16(5):442–3. 10.1046/j.1464-5491.1999.00059.x 10342346

[12] ADA. Periodontitis. Available online at: https://www.ada.org/resources/ada-library/oral-health-topics/periodontitis (cited April 14, 2024).

[13] American Academy of Periodontology. Gum disease information. Available online at: https://www.perio.org/for-patients/gum-disease-information/ (cited April 1, 2024).

[14] World Health Organization. Oral health. Available online at: https://www.who.int/news-room/fact-sheets/detail/oral-health (accessed April 10, 2024).

[15] Bramer WM Giustini D de Jong GB Holland L Bekhuis T. De-duplication of database search results for systematic reviews in EndNote. J Med Libr Assoc. (2016) 104(3):240. 10.3163/1536-5050.104.3.014 27366130 PMC4915647

[16] Ouzzani M Hammady H Fedorowicz Z Elmagarmid A. Rayyan-a web and mobile app for systematic reviews. Syst Rev. (2016) 5(1):1–10. 10.1186/s13643-016-0384-4 27919275 PMC5139140

[17] Cochrane Methods Bias. RoB 2: A revised Cochrane risk-of-bias tool for randomized trials. Available online at: https://methods.cochrane.org/bias/resources/rob-2-revised-cochrane-risk-bias-tool-randomized-trials (cited September 15, 2022).

[18] Guyatt GH Oxman AD Vist GE Kunz R Falck-Ytter Y Alonso-Coello P GRADE: an emerging consensus on rating quality of evidence and strength of recommendations. Br Med J. (2008):336(7650):924–6. 10.1136/bmj.39489.470347.AD 18436948 PMC2335261

[19] Akl E Mustafa R Santesso N Wiercioch W. GRADE handbook. (2013). Available online at: https://gdt.gradepro.org/app/handbook/handbook.html#h.rkkjpmwb6m6z (cited May 15, 2024).

[20] Badimon L Peña E Arderiu G Padró T Slevin M Vilahur G C-reactive protein in atherothrombosis and angiogenesis. *Front Immunol*. (2018) 9:430. 10.3389/fimmu.2018.00430 PMC5840191 29552019

[21] Milanesi FC Greggianin BF Dos Santos GO Toniazzo MP Weidlich P Gerchman F Effect of periodontal treatment on glycated haemoglobin and metabolic syndrome parameters: a randomized clinical trial. J Clin Periodontol. (2023) 50(1):11–21. 10.1111/jcpe.13717 36053828

[22] Jepsen S Suvan J Deschner J. The association of periodontal diseases with metabolic syndrome and obesity. Periodontal 2000. (2020) 83(1):125–53. 10.1111/prd.12326 32385882

[23] Arroniz PS Furuya MAT Gomez MA Garzón TJ Redondo CC Martínez LJA. High specificity reactive protein as a marker of periodontal disease. Oral. (2013) 14(46):1026–9. https://www.medigraphic.com/pdfs/oral/ora-2013/ora1346b.pdf

[24] D'Aiuto F Gkranias N Bhowruth D Khan T Orlandi M Suvan J Systemic effects of periodontitis treatment in patients with type 2 diabetes: a 12-month, single-centre, investigator-masked, randomized trial. Lancet Diabetes Endocrinol. (2018) 6(12):954–65. 10.1016/S2213-8587(18)30038-X 30472992

[25] Lopez NJ Socransky SS Da Silva I Japlit MR Haffajee AD. Effects of metronidazole plus amoxicillin as the only therapy on the microbiological and clinical parameters of untreated chronic periodontitis. J Clin Periodontol. (2006) 33(9):648–60. 10.1111/j.1600-051X.2006.00957.x 16856904

[26] Rooney J Wade WG Sprague SV Newcombe RG Addy M. Adjunctive effects to non-surgical periodontal therapy of systemic metronidazole and amoxicillin alone and combined. J Clin Periodontol. (2002) 29(5):410–8. 10.1034/j.1600-051X.2002.290410.x 11966932

[27] Rafiei M Kiani F Sayehmiri K Sayehmiri F Tavirani M Dousti M Prevalence of anaerobic Bacteria (*P. gingivalis*) as Major microbial agent in the incidence periodontal diseases by meta-analysis. J Dent. (2018) 19:232–42. https://pmc.ncbi.nlm.nih.gov/articles/PMC6092461/pdf/JDS-19-232.pdf PMC6092461 30175194

[28] Borges I Faveri M Figueiredo LC Mendes P Retamal-Valdes B Lira SC Different antibiotic protocols in the treatment of severe chronic periodontitis: a 1-year randomized trial. J Clin Periodontol. (2017) 44:822–32. 10.1111/jcpe.12721 28303587

[29] Muñoz Aguilera E Suvan J Orlandi M Miró Catalina Q Nart J D'Aiuto F. Association between periodontitis and blood pressure highlighted in systemically healthy individuals: results from a nested case-control study. Hypertension. (2021) 77:1765–74. 10.1161/HYPERTENSIONAHA.120.16790 33775115

[30] Chen YF Zhan Q Wu CZ Yuan YH Chen W Yu FY Baseline HbA1c level influences the effect of periodontal therapy on glycemic control in people with type 2 diabetes and periodontitis: a systematic review on randomized controlled trails. Diabetes Ther. (2021) 12:1249–78. 10.1007/s13300-021-01000-6 33481189 PMC8099950

[31] Koromantzos PA Makrilakis K Dereka X Katsilambros N Vrotsos IA Madianos PN. A randomized, controlled trial on the effect of non-surgical periodontal therapy in patients with type 2 diabetes. Part I: effect on periodontal status and glycaemic control. J Clin Periodontol. (2011) 38(2):142–7. 10.1111/j.1600-051X.2010.01652.x 21114680

[32] Greenberg AS McDaniel ML. Identifying the links between obesity, insulin resistance and β-cell function: potential role of adipocyte-derived cytokines in the pathogenesis of type 2 diabetes. Eur J Clin Investig. (2002) 32(SUPPL. 3):24–34. 10.1046/j.1365-2362.32.s3.4.x 12028372

