# Supplementary material for: Impact of subgingival periodontal treatment on systemic markers of inflammation in patients with metabolic syndrome: a systematic review of randomized clinical trials
Source: Front Oral Health. 2025 Jan 20;5:1465820. doi: 10.3389/froh.2024.1465820 (PMC11788301; doi:10.3389/froh.2024.1465820)
Supplement: Supplementary file 1 [file Table1.docx]

PubMed 20/6/2023

| Search | Query | Result |
| --- | --- | --- |
| 1 | Metabolic Syndrome[Mesh] | 38,156 |
| 2 | Metabolic Syndrome*[tiab] | 65,140 |
| 3 | Insulin Resist*[tiab] | 101,942 |
| 4 | Dysmetabolic Syndrome*[tiab] | 122 |
| 5 | Reaven[tiab] | 95 |
| 6 | Cardiometabolic Syndrome*[tiab] | 425 |
| 7 | #1 OR #2 OR #3 OR #4 OR #5 OR #6 | 155,836 |
| 8 | Subgingival Curettage[Mesh] | 2,950 |
| 9 | Gingival Curettage*[tiab] | 48 |
| 10 | Subgingival Curettage*[tiab] | 73 |
| 11 | Periodontal Epithelial[tiab] | 32 |
| 12 | Periodontal Debridement*[tiab] | 185 |
| 13 | Gingival Debridement*[tiab] | 16 |
| 14 | Subgingival Debridement*[tiab] | 184 |
| 15 | Root Plan*[tiab] | 3,671 |
| 16 | #8 OR #9 OR #10 OR #11 OR #12 OR #13 OR #14 OR #15 | 5,401 |
| 17 | #7 AND #16 | 27 |

EMBase (Ovid) 20/6/2023

Embase <1974 to 2023 June 19>

| Search | Query | Result |
| --- | --- | --- |
| 1 | exp metabolic syndrome X/ | 101878 |
| 2 | (Metabolic adj3 Syndrome*).ti,ab. | 99889 |
| 3 | (Insulin* adj3 Resist*).ti,ab. | 147562 |
| 4 | (Dysmetabolic adj3 Syndrome*).ti,ab. | 245 |
| 5 | Reaven.ti,ab. | 109 |
| 6 | (Cardiometabolic adj3 Syndrome*).ti,ab. | 744 |
| 7 | or/1-6 | 245265 |
| 8 | exp dental curettage/ | 148 |
| 9 | (Gingival adj1 Curettage*).ti,ab. | 46 |
| 10 | (Subgingival adj1 Curettage*).ti,ab. | 56 |
| 11 | (Periodontal adj1 Epithelial).ti,ab. | 34 |
| 12 | (Periodontal adj1 Debridement*).ti,ab. | 82 |
| 13 | (Gingival adj1 Debridement*).ti,ab. | 19 |
| 14 | (Subgingival adj1 Debridement*).ti,ab. | 184 |
| 15 | (Root adj1 Plan*).ti,ab. | 5881 |
| 16 | or/8-15 | 6350 |
| 17 | 7 and 16 | 18 |

Cochrane Library 20/6/2023

| Search | Query | Result |
| --- | --- | --- |
| #1 | MeSH descriptor: [Metabolic Syndrome] explode all trees | 2429 |
| #2 | (Metabolic NEAR/3 Syndrome*):ti,ab,kw | 8500 |
| #3 | (Dysmetabolic NEAR/3 Syndrome*):ti,ab,kw | 17 |
| #4 | (Cardiometabolic NEAR/3 Syndrome*):ti,ab,kw | 65 |
| #5 | (Insulin NEAR/3 Resist*):ti,ab,kw | 15759 |
| #6 | Reaven:ti,ab,kw | 4 |
| #7 | #1 OR #2 OR #3 OR #4 OR #5 OR #6 | 22303 |
| #8 | MeSH descriptor: [undefined] explode all trees | 0 |
| #9 | (Gingival NEAR/3 Curettage*):ti,ab,kw | 15 |
| #10 | (Subgingival NEAR/3 Curettage*):ti,ab,kw | 162 |
| #11 | (Periodontal NEAR/1 Epithelial):ti,ab,kw | 4 |
| #12 | (Periodontal NEAR/1 Debridement*):ti,ab,kw | 181 |
| #13 | (Gingival NEAR/1 Debridement*):ti,ab,kw | 13 |
| #14 | (Subgingival NEAR/1 Debridement*):ti,ab,kw | 142 |
| #15 | (Root NEAR/1 Plan*):ti,ab,kw | 2873 |
| #16 | #8 OR #9 OR #10 OR #11 OR #12 OR #13 OR #14 OR #15 | 3206 |
| #17 | #7 AND #16 | 32 |

CINAHL (EBSCO) 20/6/2023

| Search | Query | Result |
| --- | --- | --- |
| 1 | (MH "Metabolic Syndrome X+") | 15,019 |
| 2 | TI (Metabolic N3 Syndrome*) OR AB (Metabolic N3 Syndrome*) | 18,501 |
| 3 | TI (Dysmetabolic N3 Syndrome*) OR AB (Dysmetabolic N3 Syndrome*) | 30 |
| 4 | TI (Cardiometabolic N3 Syndrome*) OR AB (Cardiometabolic N3 Syndrome*) | 207 |
| 5 | TI (Insulin N3 Resist*) OR AB (Insulin N3 Resist*) | 22,236 |
| 6 | TI Reaven OR AB Reaven | 32 |
| 7 | S1 OR S2 OR S3 OR S4 OR S5 OR S6 | 41,953 |
| 8 | TI (Gingival N1 Curettage*) OR AB (Gingival N1 Curettage*) | 4 |
| 9 | TI (Subgingival N1 Curettage*) OR AB (Subgingival N1 Curettage*) | 6 |
| 10 | TI (Periodontal N1 Epithelial) OR AB (Periodontal N1 Epithelial) | 14 |
| 11 | TI (Periodontal N1 Debridement*) OR AB (Periodontal N1 Debridement*) | 69 |
| 12 | TI (Gingival N1 Debridement*) OR AB (Gingival N1 Debridement*) | 4 |
| 13 | TI (Subgingival N1 Debridement*) OR AB (Subgingival N1 Debridement*) | 60 |
| 14 | TI (Root N1 Plan*) OR AB (Root N1 Plan*) | 1,107 |
| 15 | S8 OR S9 OR S10 OR S11 OR S12 OR S13 OR S14 | 1,242 |
| 16 | S7 AND S15 | 5 |

LILACS (BVS Eng) 20/6/2023

| Search | Query | Result |
| --- | --- | --- |
| 1 | MH Metabolic Syndrome OR ((Metabolic$ OR Dysmetabolic OR Dismetabolic$ OR Cardiometabolic) AND (Syndrome$ OR Sindrome$)) OR ((Insulin$) AND (Resist$)) OR Reaven) AND (MH Subgingival Curettage OR ((Gingival OR Subgingival OR Gengival OR SubGengival OR Periodont$) AND (Curettage$ OR Curetage$ OR Legrado$ OR Debridement$ OR Debridamiento$ OR Desbridamento$ OR Epithelial OR Epitelial)) OR ((Root$ OR Raíz OR Raices OR Radicular) AND (Planing OR Aplainamento OR Alisado OR Aplanamiento))) [Words] | 8 |

Web of Science 20/6/2023

| Search | Query | Result |
| --- | --- | --- |
| 1 | (TS=Metabolic Syndrome OR TI=(Metabolic NEAR/3 Syndrome*) OR AB=(Metabolic NEAR/3 Syndrome*) OR TI=(Insulin NEAR/3 Resist*) OR AB=(Insulin NEAR/3 Resist*) OR TI=(Dysmetabolic NEAR/3 Syndrome*) OR AB=(Dysmetabolic NEAR/3 Syndrome*) OR TI=Reaven OR AB=Reaven OR TI=(Cardiometabolic NEAR/3 Syndrome*) OR AB=(Cardiometabolic NEAR/3 Syndrome*)) AND (TS=Subgingival Curettage OR TI=(Gingival NEAR/1 Curettage*) OR AB=(Gingival NEAR/1 Curettage*) OR TI=(Subgingival NEAR/1 Curettage*) OR AB=(Subgingival NEAR/1 Curettage*) OR TI=(Periodontal NEAR/1 Epithelial) OR AB=(Periodontal NEAR/1 Epithelial) OR TI=(Periodontal NEAR/1 Debridement*) OR AB=(Periodontal NEAR/1 Debridement*) OR TI=(Gingival NEAR/1 Debridement*) OR AB=(Gingival NEAR/1 Debridement*) OR TI=(Subgingival NEAR/1 Debridement*) OR AB=(Subgingival NEAR/1 Debridement*) OR TI=(Root NEAR/1 Plan*) OR AB=(Root NEAR/1 Plan*)) | 30 |

Scopus 20/06/2023

| Search | Query | Result |
| --- | --- | --- |
| 1 | TITLE-ABS-KEY (metabolic syndrome*) | 156,265 |
| 2 | TITLE-ABS-KEY (Insulin Resist*) | 184,986 |
| 3 | TITLE-ABS-KEY (Dysmetabolic Syndrome*) | 467 |
| 4 | TITLE-ABS-KEY (Reaven) | 158 |
| 5 | TITLE-ABS-KEY (Cardiometabolic Syndrome*) | 8,001 |
| 6 | #1 OR #2 OR #3 OR #4 OR #5 | 305,428 |
| 7 | TITLE-ABS-KEY (Subgingival Curettage*) | 992 |
| 8 | TITLE-ABS-KEY (Gingival Curettage*) | 479 |
| 9 | TITLE-ABS-KEY (Periodontal Epithelial) | 3,743 |
| 10 | TITLE-ABS-KEY (Periodontal Debridement*) | 2,536 |
| 11 | TITLE-ABS-KEY (Gingival Debridement*) | 949 |
| 12 | TITLE-ABS-KEY (Subgingival Debridement*) | 599 |
| 13 | TITLE-ABS-KEY ("Root Plan*") | 5,713 |
| 14 | #7 OR #8 OR #9 OR #10 OR #11 OR #12 OR #13 | 12,140 |
| 15 | #6 AND #14 | 63 |

Dentistry & Oral Sciences Source 20/06/2023 (EBSCO)

| Search | Query | Result |
| --- | --- | --- |
| 1 | (MH "Metabolic Syndrome") | 213 |
| 2 | TI (Metabolic N3 Syndrome*) OR AB (Metabolic N3 Syndrome*) | 277 |
| 3 | TI (Dysmetabolic N3 Syndrome*) OR AB (Dysmetabolic N3 Syndrome*) | 2 |
| 4 | TI (Cardiometabolic N3 Syndrome*) OR AB (Cardiometabolic N3 Syndrome*) | 1 |
| 5 | TI (Insulin N3 Resist*) OR AB (Insulin N3 Resist*) | 211 |
| 6 | TI Reaven OR AB Reaven | 0 |
| 7 | S1 OR S2 OR S3 OR S4 OR S5 OR S6 | 489 |
| 8 | TI (Gingival N1 Curettage*) OR AB (Gingival N1 Curettage*) | 21 |
| 9 | TI (Subgingival N1 Curettage*) OR AB (Subgingival N1 Curettage*) | 17 |
| 10 | TI (Periodontal N1 Epithelial) OR AB (Periodontal N1 Epithelial) | 30 |
| 11 | TI (Periodontal N1 Debridement*) OR AB (Periodontal N1 Debridement*) | 94 |
| 12 | TI (Gingival N1 Debridement*) OR AB (Gingival N1 Debridement*) | 25 |
| 13 | TI (Subgingival N1 Debridement*) OR AB (Subgingival N1 Debridement*) | 175 |
| 14 | TI (Root N1 Plan*) OR AB (Root N1 Plan*) | 2,303 |
| 15 | S8 OR S9 OR S10 OR S11 OR S12 OR S13 OR S14 | 2,588 |
| 16 | S7 AND S15 | 10 |
